# In Vivo Performance of Visual Criteria, Laser-Induced Fluorescence, and Light-Induced Fluorescence for Early Caries Detection

**DOI:** 10.3390/diagnostics13203170

**Published:** 2023-10-11

**Authors:** Antonis Perdiou, Aurora Doris Fratila, Ruxandra Sava-Rosianu, Vlad Tiberiu Alexa, Dacian Lalescu, Daniela Jumanca, Atena Galuscan

**Affiliations:** 1 Faculty of Dental Medicine, “Victor Babeş” University of Medicine and Pharmacy, Eftimie Murgu Sq. No. 2, 300041 Timisoara, Romania; antonis.perdiou@umft.ro (A.P.); sava-rosianu.ruxandra@umft.ro (R.S.-R.); vlad.alexa@umft.ro (V.T.A.); jumanca.daniela@umft.ro (D.J.); galuscan.atena@umft.ro (A.G.); 2 Faculty of Dental Medicine, Ludwig-Maximilian-University Munich, Goethestraße 70, 80336 München, Germany; 3 Translational and Experimental Clinical Research Center in Oral Health (TEXC-OH), 14A Tudor Vladimirescu Ave., 300173 Timisoara, Romania; 4 Faculty of Food Engineering, Banat’s University of Agricultural Sciences and Veterinary Medicine, King Michael I of Romania from Timișoara, Calea Aradului No. 119, 300645 Timișoara, Romania; lalescu@usab-tm.ro

**Keywords:** intraoral scanners, oral health, fluorescence, dental caries, digital dentistry, diagnosis

## Abstract

This study aims to compare the diagnostic reliability of ICDAS-II visual criteria, light-induced fluorescence (using the VistaCam iX, Dürr Dental, Bietigheim-Bissingen, Germany), and laser-induced fluorescence (using the DIAGNOdent Pen, KaVo, Biberach, Germany) on occlusal caries. Permanent and temporary molars were selected according to the inclusion criteria. Out of 160 teeth that met the inclusion criteria, 139 were chosen and examined by two previously trained and calibrated examiners. The kappa value was 0.95 for both VistaCam iX and DIAGNOdent Pen. Results from visual examination and the readings of the two fluorescence devices were computed, lesions being divided into non-cavitated, enamel lesions, and lesions extended to dentin. All statistical analyses were performed using R (version 4.2.2). Spearman’s rank correlation was computed to assess the relationship between the scores of diagnostics reliabilities of the three methods mentioned above. There was a positive, statistically significant Spearman’s rank correlation coefficient, ρ = 0.25, between VistaCam iX and ICDAS II, and a positive, not statistically significant Spearman’s rank correlation coefficient, ρ = 0.11, between DiagnoDent Pen and ICDAS II. Considering the temporary teeth, there was a positive, statistically significant Spearman’s rank correlation coefficient, ρ = 0.52, between VistaCam iX and DiagnoDent Pen; a positive, statistically significant Spearman’s rank correlation coefficient, ρ = 0.35, between VistaCam iX and ICDAS II; and the lowest, not statistically significant Spearman’s rank correlation coefficient, ρ = 0.16, between DiagnoDent Pen and ICDAS II. Conclusions: In conclusion, ICDAS II and light-induced fluorescence are better diagnostic methods than the laser-induced fluorescence devices for detecting occlusal caries. Clinical Significance: This study may support clinicians in selecting the most efficient tool for diagnosing carious lesion in the earliest stages possible. Furthermore, such technologies raise the availability for more preventive approaches, as opposed to invasive procedures.

## 1. Introduction

Although dental caries is a preventable non-communicable disease, reports show that oral health has not improved in the past 25 years [1]. In 2021, the most recent WHO resolution underlined the need to improve oral health worldwide [2]. For good oral health, it is necessary, among others, to detect and treat dental caries as early as possible [3]. Oral health is reckoned to contribute to the general well-being of individuals, thus maintaining a healthy oral cavity and teeth free of pathological problems such as caries or periodontal disease is an important objective that needs constant improvement in permanent and deciduous dentition [4]. Therefore, the role of temporary dentition should not be underestimated. The role of primary dentition in humans is well known: temporary teeth contribute to the development of the upper and lower jaw; they serve as space maintainers before the eruption of permanent teeth until they reach their natural exfoliation period. Among others, mastication is one of the essential functions of temporary teeth [5]. In children, tooth decay is one of the most common diseases worldwide [6]. Left untreated, it often results in deciduous teeth’ extraction, compromising the individual’s well-being. Therefore, early diagnosis and evaluation of white spot lesions and signs of demineralization are essential for the early treatment and prevention of dental caries in both temporary and permanent dentition.

In recent years, several methods have been sought to improve the detection of carious lesions. Until now, most dental healthcare providers focused primarily on clinically visible lesions while diagnosing. The visual and tactile methods, which include examining the tooth surface by direct vision or with the help of a probe, are the most used diagnostic methods for dental caries at the moment [7]. One of the most challenging aspects when diagnosing dental caries is quantifying clinical observations into an objective numerical interpretation. One attempt to solve this problem was the development of the DMFT index, which quantifies the oral health status of an individual by the number of decayed (D), missing (M), and filled (F) teeth. This index was unsatisfactory and, in many cases, did not provide information about the severity of the disease or the need for extensive dental care [7]. Moreover, without radiographs, the DMFT index undervalued the carious lesions 44% of the time [8]. In order to help dentists properly diagnose dental caries, a universal scoring system called ICDAS II (The International Caries Detection and Assessment System) was established [9]. The system coding relies strictly on visual inspection of the lesion [10,11]. ICDAS provides up to 43% more information than the DMFT/dmft index, according to Coelho (2020) [12]. ICDAS II classifies lesions as active or inactive [10] and helps the dental professional decide on prognosis and treatment planning [11]. Observing the activity of the carious lesion can provide a better understanding of whether a preventive or therapeutic method can be used. An in vivo study conducted by Ferreira et al. (2012) in Puerto Rico demonstrated that an intervention should be made earlier (sealing the pits and fissures) for carious lesions that score ICDAS 3 and 4, whereas lesions with scores ICDAS 1 and 2 should be followed in their progress [13]. Alongside visual examination, dentists can use other methods of evaluating dental decay. Laser fluorescence-based devices measure the emitted fluorescent infrared light and show the result in whole numbers. It is based on the principle that chromospheres in the dental enamel and dentin cause auto-fluorescence, which is reduced by demineralization. Chromophores, like porphyrins, in carious lesions and bacteria also cause fluorescence, which can be quantified and measured by subtracting the fluorescence of a sound tooth surface from that of a carious tooth surface [14,15]. The presence of blood or other fluids in the oral cavity influences fluorescence readings. Therefore, the teeth surfaces must be thoroughly dried beforehand. To enhance patient compliance, an intraoral camera was designed to save and display clinical pictures of patients’ teeth. The long-term “monitoring” of incipient lesions can also be improved since the images can be accessed at any time [16]. To the authors’ knowledge, there are several in vitro studies in the literature, but very few in vivo studies comparing visual examination, laser-induced fluorescence, and light-induced fluorescence in detecting occlusal caries in posterior permanent and temporary teeth. Given the modern approach in cariology, where initial caries is considered reversible by different infiltration techniques, it is very important to be able to detect those lesions as early as possible. The study seeks to evaluate the extent to which laser fluorescence and light-induced fluorescence devices can enhance the outcomes derived from visual examinations and the identification of early lesions. It aims to assess the diagnostic reliability of both laser-induced fluorescence and light-induced fluorescence in primary and permanent teeth with occlusal caries.

## 2. Materials and Methods

### 2.1. Ethical Approval

The proposal of this study was approved by the ethics committee of the Victor Babes University of Medicine and Pharmacy, Timisoara (Nr.08/26.02.2021), and informed consent was obtained from all patients before the initiation of the study.

### 2.2. Study Design

**Figure 1:**
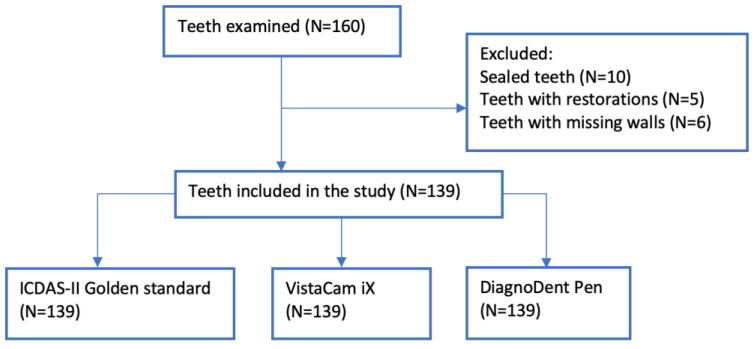
Flow diagram of the selection process.

**Figure 2:**
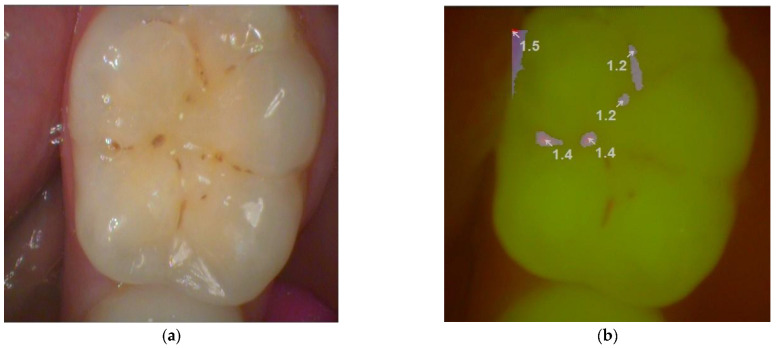
Picture of a permanent molar taken by light-induced fluorescence device (**a**) with magnifying lens, (**b**) using the fluorescent mode of VistaCam iX. (Dürr Dental, Germany).

This study was conducted on patients aged 7 to 17, referred to the department of Dental Prevention, Community Dentistry, and Oral Health (University of Medicine and Pharmacy, Victor Babes, Timisoara, Romania). Two calibrated dentists performed the clinical examinations. The inter-examiner reliability was tested using the kappa value, a value used to compare two examiners with different score ranges. The kappa value for this study was 0.95, considered almost perfect agreement according to the kappa scores developed by Landis and Koch (1977) [17]. Inclusion criteria addressed patients with signs of pit and fissure caries in at least one permanent or temporary tooth in the posterior region. A clinical examination of each tooth was carried out under adequate lighting after cleaning the tooth surfaces. The samples used in the pilot study were not included in the main study. One-hundred-and-thirty-nine (139) teeth were included in the study: ninety-seven permanent posterior teeth and forty-two primary posterior teeth. The teeth were intact or had incipient and inconspicuous caries with or without color changes. Teeth showing occlusal restorations, enamel hypoplasia, hypomineralization or structural defects, and pulp necrosis were excluded (Figure 1). In comparison, the two calibrated dentists examined the teeth with a laser fluorescent pen (DIAGNOdent, Kavo, Biberach, Germany) and an intraoral fluorescent camera using the fluorescent head of a light-induced fluorescence device (Dürr Dental, Germany) (Figure 2).

### 2.3. Visual Examination Using the International Caries Detectoin and Assesment System (ICDAS II)

Visual examination was performed using the ICDAS II caries codes. Occlusal surfaces of the teeth were cleaned from plaque and debris using water spray and cotton pellets if necessary. Dental explorers were not used for examination. Occlusal caries was scored according to the ICDAS-II System:

Code 0: Sound tooth surface: No evidence of caries after 5 s of air drying.

Code 1: First visual change in enamel: Opacity or discoloration (white or brown) is visible at the entrance to the pit, and a fissure is seen after prolonged air drying.

Code 2: Distinct visual change in enamel visible when wet; lesion must be visible when the tooth is dry.

Code 3: Localized enamel breakdown (without clinical visual signs of dentinal involvement) seen when wet and after prolonged drying.

Code 4: Underlying dark shadow from dentine.

Code 5: Distinct cavity with visible dentine.

Code 6: Extensive (more than half of the tooth surface) distinct cavity with visible dentine.

### 2.4. Examination Using Laser-Induced Fluorescence

A laser fluorescence device (DIAGNOdent Pen, KaVo, Biberach, German) was used for the measurements. A sound-appropriate dental surface of a central or lateral incisor was selected for calibration. This allowed determining a value for each occlusal surface. The highest laser fluorescence value was recorded after three readings for each occlusal surface [18,19].

### 2.5. Examination Using Light-Induced Fluorescence

**Figure 3:**
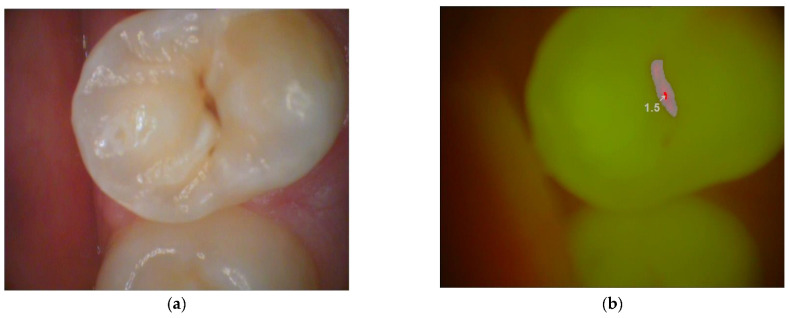
Picture of a permanent premolar taken by light-induced fluorescence device. (**a**) With magnifying lens, (**b**) using the fluorescent mode of VistaCam iX. (Dürr Dental, Germany).

The examination using light-induced fluorescence (VistaCam iX, Dürr Dental, Bietigheim-Bissingen, Germany) consisted of a camera handpiece with two interchangeable lenses so that it could be used not only with the fluorescence attachment for detecting caries but also as a conventional intra-oral camera, and it was connected to a laptop by a USB cable (Figure 3). The patient was positioned in dorsal decubitus on the chair. Cotton rolls were placed in the mouth and thoroughly air dried from the unit. A light-induced fluorescence device was mounted on a calculator, and all the lights were turned off. We first took pictures of the teeth using the fluorescent head and then the white light head. The DBSWIN 5.17 evaluation software was also used to store and visualize data. Thus, communication with the patient and therapy planning was more effective. The light-induced fluorescence device (Durr Dental, Bietigheim-Bissingen Germany) scoring was 0–1.2 = sound tissue; 1.3–1.5 = enamel caries; and >1.5 = dentine caries [11,20]. In order to evaluate the diagnostic reliability of the systems mentioned above, the measurements were coded with 0, 1, and 2 (see Table 1). The Wilcoxon signed-rank test was employed to identify statistical differences in the diagnostic reliability scores among the three methods. Spearman’s rank correlation was computed to assess the relationship between the scores of diagnostics reliability of the three methods mentioned above. All statistical analyses were performed using R (version 4.2.2).

The objective was to examine the degree of association between these diagnostic methods in the context of evaluating occlusal caries in primary and permanent teeth. The statistical analysis aimed to quantify the relationships and provide insights into the interplay and consistency among the tools in detecting and diagnosing dental lesions.

## 3. Results

Table 2 presents the outcomes obtained through the application of three diagnostic methods. In the visual examination (ICDAS), 38.85% (*N* = 54) of examined teeth received a score of 0, whereas 55% (*N* = 80) scored 1, and 3.6% (*N* = 5) scored 2. The laser-induced fluorescence device examination found 79.78% (*N* = 97) of the teeth scoring 0, 10.7% (*N* = 14) scoring 1, and 20.14% (*N* = 28) scoring a 2. Conversely, the examination using the light-induced fluorescence device revealed that 37.41% (*N* = 52) of the teeth scored 0, whereas 55.40% (*N* = 77) scored 1, and 7.19% (*N* = 10) scored 2. For permanent teeth, the visual examination (ICDAS) results showed that 35.02% (*N* = 34) of the teeth scored 0, 63.92% (*N* = 62) scored 1, and 1.03% (*N* = 1) scored 2. The laser-induced fluorescence device examination found 68.04% (*N* = 66) of the teeth scoring 0, 12.37% (*N* = 12) scoring 1, and 29.59% (*N* = 19) of the teeth 2. Lastly, the light-induced fluorescence device examination results showed that 31.96% (*N* = 31) of the teeth scored 0, 59.79% (*N* = 59.79) scored 1, and 8.25% (*N* = 8) scored 2. In the case of temporary teeth, the visual examination (ICDAS) results showed that 47.62% (*N* = 20) of the teeth scored 0, 42.86% (*N* = 20) scored 1, and 9.52% (*N* = 4) scored 2. The laser-induced fluorescence device examination found 73.81% (*N* = 31) of the teeth scoring 0, 4.76% (*N* = 2) scoring 1, and 21.43% (*N* = 9) of the teeth 2. Lastly, the light-induced fluorescence device examination results showed that 50.00% (*N* = 21) of the teeth scored 0, 45.24% (*N* = 19) scored 1, and 4.76% (*N* = 2) scored 2.

**Figure 4:**
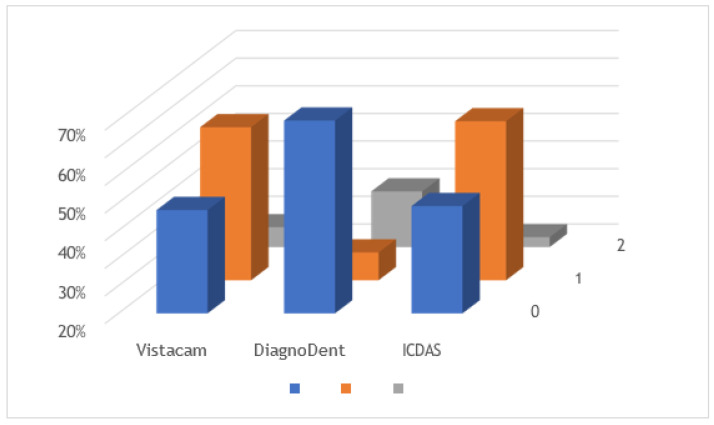
Comparison graph of all teeth using the three diagnostic methods.

Figure 4 shows the comparison between the three methods, taking into account the total sample size. The Wilcoxon signed-rank test showed no statistically significant differences between ICDAS II scores and light-induced fluorescence device scores (*p* = 0.4) and marginally significant differences between ICDAS II scores and laser-induced fluorescence device scores (*p* = 0.1).

**Figure 5:**
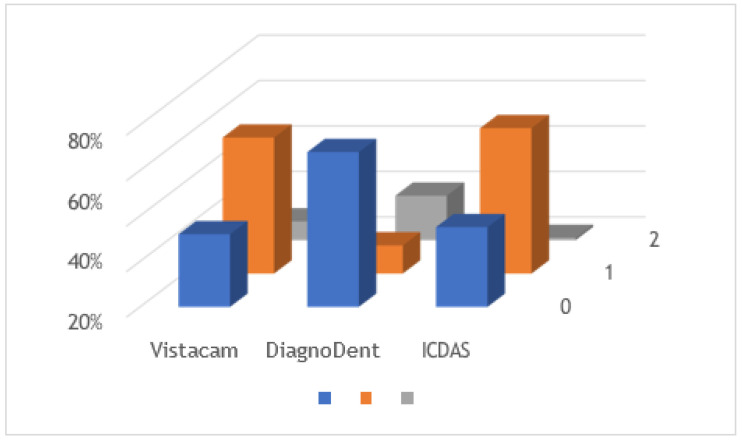
Comparison graph of permanent teeth using the three diagnostic methods.

In the context of permanent dentition, statistical analysis revealed a lack of significant differences between ICDAS II scores and scores obtained through light-induced fluorescence devices (*p* = 0.18). However, marginal significance was observed in the comparison between ICDAS II scores and those generated by laser-induced fluorescence devices (*p* = 0.15), as depicted in Figure 5.

**Figure 6:**
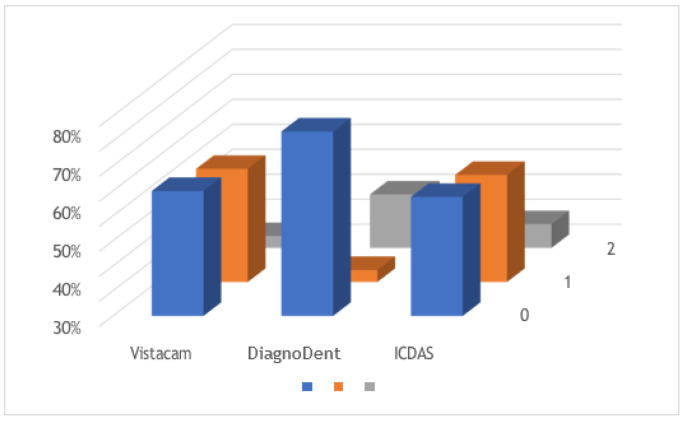
Comparison graph of temporary teeth using the three diagnostic methods.

Considering only primary teeth, there were no statistically significant differences between ICDAS-II scores and light-induced fluorescence device scores (*p* = 0.7) and between ICDAS-II scores and laser-induced fluorescence device scores with (*p* = 0.4) (Figure 6).

**Figure 7:**
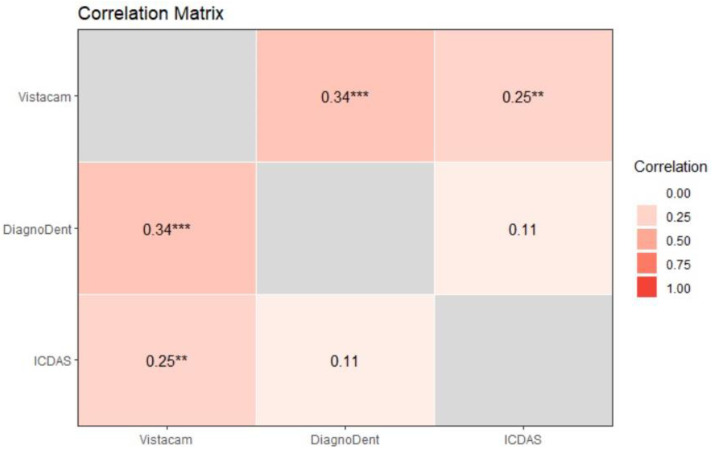
Correlation Matrix of all the examined teeth. Two stars (‘**’), Three stars (‘***’) denote that the corresponding variable is significant at 5%, 1% level, respectively. Absence of star denotes no significant variable.

For all the examined teeth, a Spearman’s rank correlation matrix was computed. A statistically significant positive Spearman’s rank correlation was found between light-induced fluorescence and laser-induced fluorescence (ρ = 0.34, *p* < 0.001, 95% C.I. 0.17 to 0.48). A smaller but statistically significant positive Spearman’s rank correlation was observed between the light-induced fluorescence device and ICDAS II (ρ = 0.25, *p* = 0.007, 95% C.I. 0.08 to 0.40). In contrast, the positive Spearman’s rank correlation between the laser-induced fluorescence device and ICDAS was 0.11 (*p* = 0.215), with a 95% C.I. from −0.07 to 0.27, indicating a lack of statistical significance (Figure 7).

**Figure 8:**
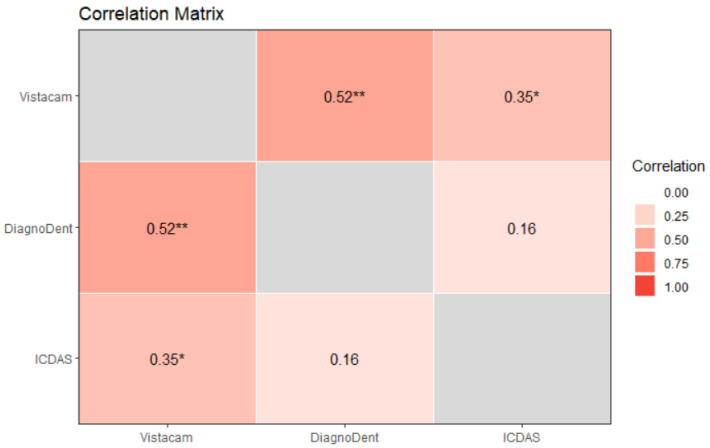
Correlation matrix on temporary teeth. One star (‘*’), Two stars (‘**’) denote that the corresponding variable is significant at 10%, 5% level, respectively. Absence of star denotes no significant variable.

The same correlation matrix was calculated, taking into account only temporary teeth. Light-induced fluorescence and laser-induced fluorescence had a moderate positive correlation with ρ = 0.52, confidence level 95% (0.24, 0.71), and *p* = 0.001, being statistically significant. Light-induced fluorescence and ICDAS experienced a moderate positive correlation with ρ = 0.35, confidence level 95% (0.04, 0.60), and *p* = 0.045 being statistically significant. Laser-induced fluorescence and ICDAS II had the lowest correlation (ρ = 0.16), and there was no statistical significance (*p* = 0.296) (Figure 8).

**Figure 9:**
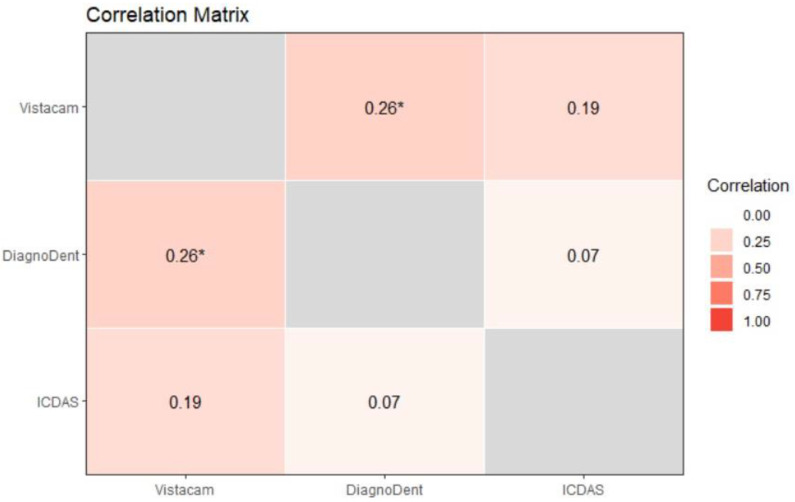
Correlation matrix of permanent teeth. One star (‘*’) denote that the corresponding variable is significant at 10% level. Absence of star denotes no significant variable.

For permanent teeth, correlation showed that light-induced fluorescence devices and laser-induced fluorescence devices exhibited a mild correlation (ρ = 0.26) and *p* = 0.034, which made it statistically significant. Light-induced fluorescence devices and ICDAS II had a mild correlation but were statistically insignificant, *p* = 0.115. Laser-induced fluorescence and ICDAS II had almost no correlation (ρ = 0.07) and *p* = 0.487, as shown in Figure 9.

## 4. Discussion

As the medical community has moved from an invasive to a minimally invasive philosophy of accomplishing treatments, detecting early dental caries and demineralization have become essential [21]. The visual examination of dental caries has proven effective, mainly in the more advanced stages of caries. For this reason, more studies are evaluating innovative methods of caries identification and quantification, so that dental practitioners can diagnose dental caries and demineralization in the early stages, thus contributing to preventive dental care and oral health [22]. When all the examined teeth were accounted for, a strong correlation was observed between light-induced fluorescence and laser-induced fluorescence as well as between light-induced fluorescence and visual examination. The correlation between laser-induced fluorescence and visual examination was not statistically significant. All of those mentioned above were calculated at a confidence level of 95%. Akarsu et al. found in their study between visual examination and a combined examination (visual examination and examination with laser fluorescence) on extracted teeth with non-cavitated lesions that combined examination yielded better results than visual examination alone [23]. Shi et al. concluded in another study using laser-induced fluorescence devices and standard radiographs on extracted posterior teeth that laser-induced fluorescence devices better identified occlusal caries than a standard radiograph. They also noted that one of the disadvantages of the laser-induced fluorescence device was that the occlusal surface to be examined had to be thoroughly dried before using the light fluorescence device [24]. However, Rodriguez et al. found out that the combination between ICDAS and BW had the best results with the only problem being the reproducibility of the BW outcome between ICDAS, laser fluorescence, and bitewing X-ray (BW). According to Landis and Koch, the k value of the inter-examiner evaluation was 0.5, which is a moderate value [25]. In another in vitro study, Jablonski et al. compared light-induced fluorescence devices, laser-induced fluorescence devices, and ICDAS in occlusal caries, with the highest correlation (r = 0.84) being between light-induced fluorescence device and ICDAS as it is in this study as well [26]. The null hypothesis was that all three methods would have the same results. However, this hypothesis held true solely for light-induced fluorescence devices. Occlusal caries is the most common form of caries in children, adolescents, and young adults, presenting 75% of the total caries detection, as shown by a study of Lussi et al. [27]. Also, the occlusal surface is the most challenging surface to examine reliably for caries detection [18,28]. To the authors’ knowledge, very few studies on the clinical validity of light-induced fluorescence device on temporary dentition. A study from Ahrani et al. on extracted temporary teeth showed that light-induced fluorescence device was statistically significant, with the golden standard being the histological examination for caries in the approximal surfaces [29]. ICDAS-II can be a difficult task for beginners and can cause over-treatment. A recent study by Qudeimant et al. showed that there were evident inconsistencies between examiners for initial caries (ICDAS < 2) and accordance for extensive carious lesions with ICDAS > 3 [30]. Mazur et al. [31] observed poor agreement.

## 5. Conclusions

Based on the study’s objectives, it was established that incorporating light-induced fluorescence as a supplementary method alongside visual examination enhanced the precision of caries detection. However, the study revealed a tendency for laser-induced fluorescence to underestimate non-cavitated lesions, inaccurately categorizing them as healthy tissue. The limitations of relying solely on visual examination were also highlighted due to the subjective nature among professionals. Notably, ICDAS and light-induced fluorescence emerged as superior diagnostic methods compared to laser-induced fluorescence in identifying occlusal caries. Future investigations should focus on evaluating the impact of intervals in laser-induced fluorescence devices on the outcomes of visual examinations.

## Figures and Tables

**Table 1:** Coding for the statistical tests.

Code	ICDAS-II	DiagnoDent	VistaCam IX
0	0	0–14	0–1.2
1	1, 2, and 3	15–20	1.3–1.5
2	4, 5, and 6	21–99	>1.5

**Table 2:** Results of the diagnostic instruments for all teeth.

	0	1	2
		All teeth	
ICDAS II	38.85% (*N* = 54)	57.55% (*N* = 80)	3.6% (*N* = 5)
VistaCam Ix	37.41% (*N* = 52)	55.40% (*N* = 77)	7.19% (*N* = 10)
DIAGNOdent pen	69.78% (*N* = 97)	10.07% (*N* = 14)	20.14% (*N* = 28)
Permanent teeth			
ICDAS II	35.02% (*N* = 34)	63.92% (*N* = 62)	1.03% (*N* = 1)
Vistacam Ix	31.96% (*N* = 31)	59.79% (*N* = 58)	8.25% (*N* = 8)
DIAGNOdent pen	68.04% (*N* = 66)	12.37% (*N* = 12)	19.59% (*N* = 19)
	Temporary teeth		
ICDAS II	47.62% (*N* = 20)	42.86% (*N* = 18)	9.52% (*N* = 4)
Vistacam Ix	50.00% (*N* = 21)	45.24% (*N* = 19)	4.76% (*N* = 2)
DIAGNOdent pen	73.81% (*N* = 31)	4.76% (*N* = 2)	21.43% (*N* = 9)

## Data Availability

The data presented in this study are available on request from the corresponding author. The data are not publicly available due to patient privacy.
